# Preliminary studies of hair follicle regeneration by injections of epidermal stem cells and dermal papilla cells into nude mice

**DOI:** 10.1007/s10561-020-09825-4

**Published:** 2020-03-11

**Authors:** Mingsheng Zhang, Yan Ye, Pin Zhao, Liming Bai, Xinping Li

**Affiliations:** 1 Department of Physical Medicine and Rehabilitation, Guangdong Geriatric Institute, Guangdong Academy of Medical Sciences and Guangdong Provincial People’s Hospital, Guangzhou, 510080 China; 2 The Second People’s Hospital of Foshan, Affiliated Foshan Hospital of Southern Medical University, Guangzhou, 528000 China; 3 Guangzhou Huayin Medical Laboratory Center, Guangzhou, 510515 China

**Keywords:** Hair follicle, Regeneration, Epidermal stem cells, Dermal papilla cells

## Abstract

The ultimate goal of organ regenerative therapy is to reproduce fully functional organs to replace which have been damaged as a result of diseases or injury. Although several studies claimed that using different types of cells in some animal models promote hair follicles regeneration, more researches can be done to develop a sufficient and efficient protocol to induce hair generation from different animal models. In this study, we investigated the therapeutic potentials for hair follicle formation by injecting a mixture of epidermal stem cells and dermal papilla cells. Those cells were isolated and culture-expanded. Then we randomly allocated 8 nude mice into two groups. The experiment group received an injection of a mixture that containing of epidermal stem cells and dermal papilla cells. The control group received injection of keratinocyte serum-free medium. The hair follicles regeneration was observed and the injection area was harvested for HE staining. 14 day later, the regenerated hair shafts were observed and HE staining indicated that the newly hair follicle formed the correct structures in experiment group. Furthermore, the mixture injection induced a regular and multilayered stratified epidermis and the epidermis contained of hair follicle-likes structures. Our data showed that injection of a mixture of epidermal stem cells and dermal papilla cells could induce hair follicles regeneration and well-ordered epidermis formation. This study emphasized that the rearrangement of the interactions during seed cells and the niches of the seed cells is essential and necessary for tissue-engineered construct success.

## Introduction

Many novel therapeutic strategies have been made in accelerating wound healing, but the results are not entirely satisfactory because of scar formation and lack of normal function appendages. Furthermore, the hair coat can severely affect thermoregulation and social communication. Thus, the ultimate goal of wound treatment is to achieve a flawless skin regeneration by reducing scar formation and recovering the anatomy of skin and its normal function (Fu and Li 2009).

Advancing in stem cells research, several studies have shown that stem cell-based therapies are attractive candidates in regenerative medicine to treat skin wounds (Zhang et al. 2012; Yoo and Lim 2009). As one kind of tissue-specific stem cells, epidermal stem cells (ESCs) are essential elements for maintaining the structure of skin tissue and homeostasis (Li et al. 2017). And several studies claimed that Hair follicle regeneration was observed in some kinds of full thickness wound models after the transplantation of a mixture or skin substitutes containing ESCs (Wang et al. 2016). Interestingly, the data from recent studies (Ito et al. 2007; Jiang et al. 2010) suggested that cutaneous wounds could induce follicular neogenesis in mice models with full thickness wound, meaning that the use of ESCs-based therapies in wound healing models represents a useful strategy for the treatment of skin injuries without non-scarring alopecias, and there needs more information on treatment strategies using ESCs for hair preservation in different models with hair loss.

Hair follicle is made of a permanent region which contains dermal papilla (DP) cells, hair matrix, and differentiated epithelial cells (Schmidt-Ullrich and Paus 2005). After hair follicle morphogenesis, many kinds of stem cells are maintained in different regions, such as epidermal stem cells in the bulge region of follicle (Asakawa et al. 2012). Generally the epithelial-mesenchymal interactions are crux for normal development of the hair follicle (Kishimoto et al. 1999). The interactions between epidermal stem cells and mesenchymal cells mediate hair growth and hair cycle, which is depends on the activation of DP cells (Toyoshima et al. 2012). When DP cells deriving from mesenchyme provide unique and critical signals to ESCs, ESCs undergo a highly coordinated and stepwise program of differentiation to form hair follicle. Thus, to achieve hair follicle regeneration, it is thought to be essential to regenerate the interactions. However, a strategy to interactive signaling between DP cells and ESCs in vitro did not regenerate new hair follicles (HFs) (Qi et al. 2008). Fortunately, injection of a mixture of dermal papilla and epithelial cells could induce hair growth in nude mice (Nilforoushzadeh et al. 2017). We all know that ESCs are capacity of differentiating into epithelial cells, and it remains to be determined whether the mixture of DP cells and ESCs can regenerate hair follicles though intracutaneous injection.

Here, we investigated the therapeutic potentials of a mixture for hair follicle formation. In our current study, we choose ESCs as seed cells, and then prepared the mixture which contained DP cells and ESCs. After injected the mixture into the dorsal cranial region of nude male mice for 14 days, the tissue sections were sampled for histological examination. This may be a promising strategy for the repair and regeneration of skin wound and its appendages.

## Methods and materials

### Animals

SD rats and Nude male mice at 8–9 weeks of age were purchased from the Animal Laboratory of Sun Yat-sen University. The animal room was approximately maintained at 25 °C with 50 ± 10% humidity, using a 12-h light/dark cycle. They were fed by the common diet and allowed free access to tap water.

### Isolation and Culture of EPCs and DP cells

Dermal papilla (DP) cells were isolated from HFs as described in previous research (Kishimoto et al. 2009) and were cultured in DMEM (Sigma, St. Louis, MO) with 10% fetal bovine serum (FBS, Hyclone, Logan, UT) and 1% P/S from passage 1–2.

Isolation of epidermal stem cells from the rat skin tissue was established in previous research (Zhang et al. 2013), the cells were cultured in keratinocyte serum-free medium (K-SFM) supplemented with 5 ng/ml epidermal growth factor and 50 mg/ml bovine pituitary extract (Gibco). Cells between passages 3 and 4 were used for experiments.

### Mixture Transplantation

Cultured ESCs were labeled using DiI (Sigma) to determine whether hair formation in complexes originated from seeded cells. We mixed 1 × 10^6^ DP cells with 1 × 10^6^ ESCs in 10 μl K-SFM. 30 min later at 37 °C, the mixture or K-SFM was implanted into the epidermal layer or corium layer of nude male mice using 1 ml syringe with 18 G needle. 16 mice were injected with the mixture, and those mice was divided at random into the experiment group and the control group. After transplantation for 7 days, the epithelial cyst at the injection site was cut off. When mice were sacrificed after the mixture was injected for 7 days or 14 days, the skin samples where the mixture was injected were harvested for histological analysis.

### Histological examination

Samples were fixed in 10% formalin, prepared as paraffin blocks, sectioned at 6 μm, and stained with H&E. In brief, paraffin sections of the skin were routinely dewaxed, rehydrated and washed by running tap water. Firstly, paraffin sections were stained with the hematoxylin for 2.5 min, and then washed in running tap water. After differentiation and rewashing, sections were counterstained with the eosin for 10 s, then dehydrated with graded ethanols and mounted with neutral balsam. We observed them and took pictures under the microscope (Leika, Germeny).

### Scanning electron microscope (SEM)

The hair shafts were dehydrated in 100% ethanol. After coating with platinum, the samples were examined with a Hitachi S-4700 SEM (Hitachi High-Tech, Tokyo, Japan) at 15 kV. SEM observations were performed mainly for the surfaces of the lower portion of the hair shafts.

## Results

### Hair induction after implantation of ESCs and DP cells complex

**Fig. 1:**
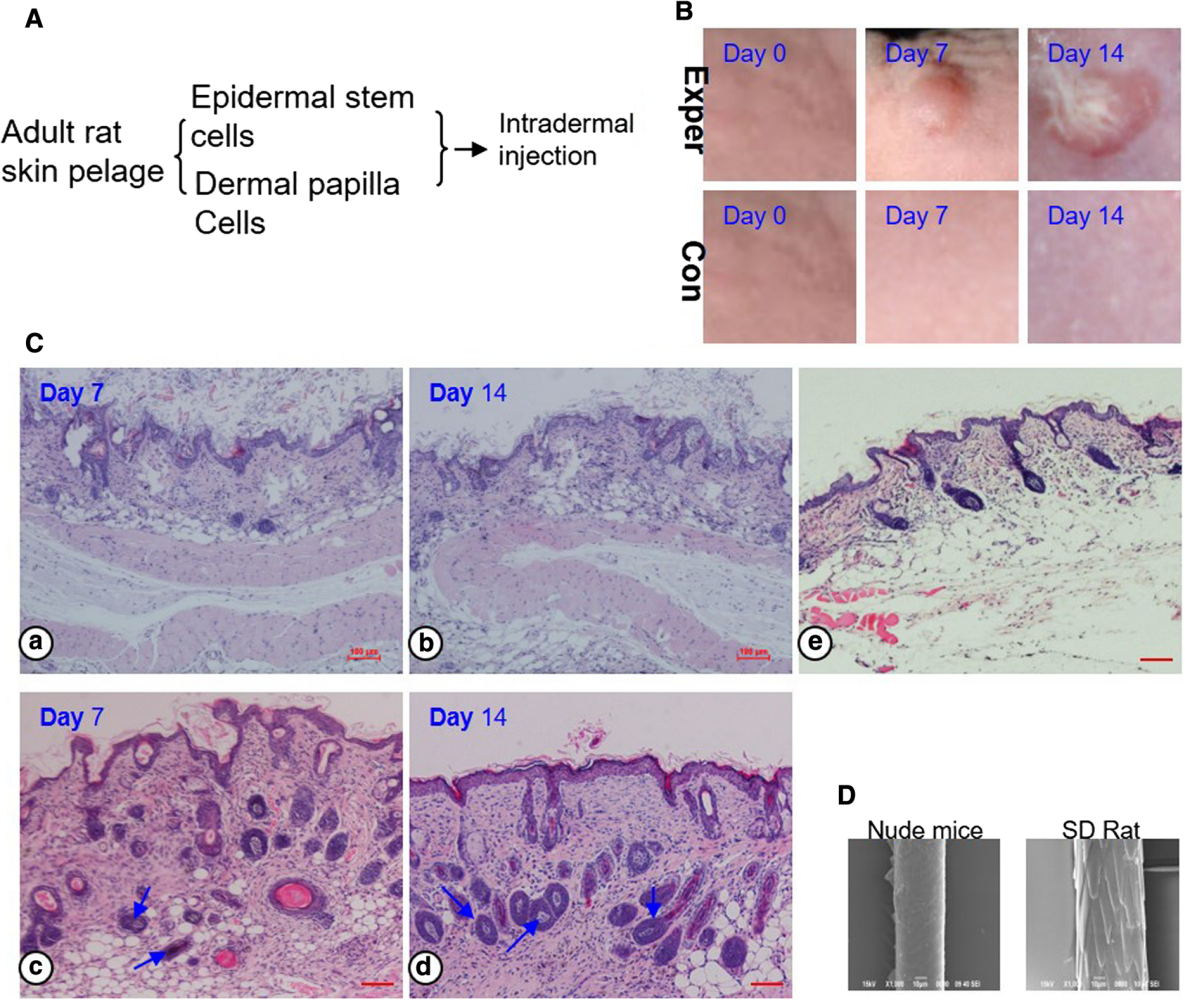
Methods and results for hair follicle regeneration. **A** Schematic representation of methods for the generation and transplantation of a mixture. **B** Macro-morphological observation of the hair. The hair shafts erupted out of the skin at day 14 in the experiment group (upper). **C** HE staining analyses of the bioengineered follicles. The green arrowhead indicates hair matrix. The red arrowhead indicates ORS and IRS. Contol (**a**, **b**) versus experiment group (**c**, **d**), normal skin tissue form the nude mice (**e**). Scale bars, 50 µm. (**D**) Newly hair was analysed by SEM. Compare to the normal hair of SD rat. (Color figure online)

All of animals kept healthy throughout the total experiment period. At 7th day after implantation, the epithelial layer formed an epithelial cyst. 14 days later, if the epithelial cyst was slice off, the eruption of hair shafts was observed, and the hair follicle did not grow out of the skin without slicing off the cyst (Fig. 1B). HE staining revealed that newly hairs could be observed. The newly hair follicles seem to be histologically correct which contained the outer root sheath (ORS), inner root sheath (IRS), hair bulb and hair matrix. The generated DP located at the distal end of the bioengineered hair follicle and was surround by hair matrix (Fig. 1C). Compare with the hairs from SD mouse, the bioengineered hair follicles were smaller in size (Fig. 1D).

### Bioengineered skin formation after implantation of ESCs and DP cells complex

**Fig. 2:**
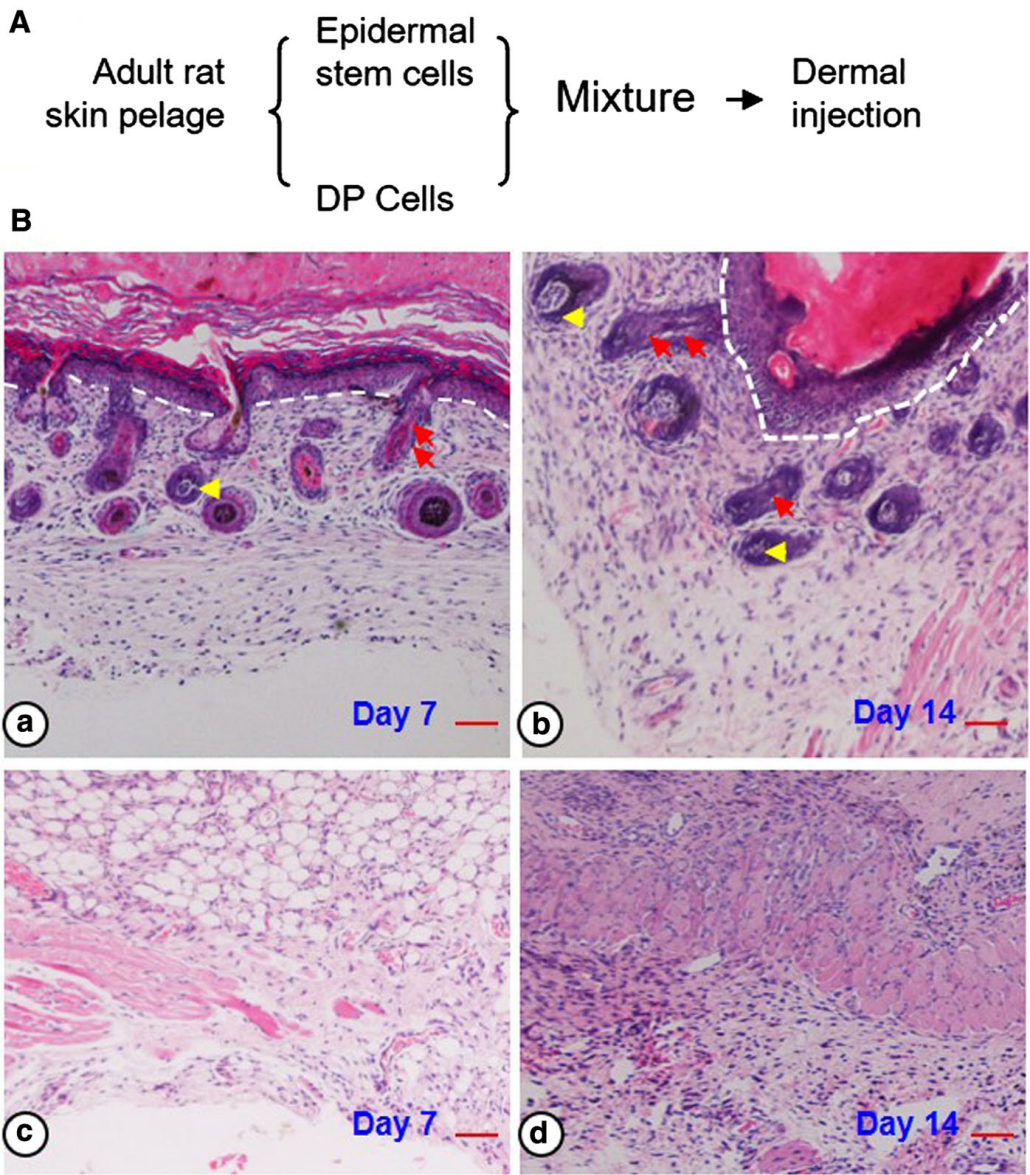
Histology of skin reconstruct in nude mice. White broken line indicated dermal-epidermal detachment. Yellow arrows, hair matrix. Red arrows, ORS and IRS. The experiment group (**A**, **B**) versus the control group (**C**, **D**). Scale bars, 50 µm. (Color figure online)

HE staining revealed that the mixture, which contain ESCs and DP cells (Fig. 2A), was induced to form 4–6 epidermal layers with sebaceous glands and hair follicles. However, no new epidermis and its appendages were observed in the control group. The mixture could form the correct structures of hair follicle which was composed of the IRS, ORS, hair bulb and hair matrix (Fig. 2B).

## Discussion

In the current study, after injection of a mixture of DP cells and ESCs, the hair follicles regeneration was observed, and the hair follicle had the correct structures. Meanwhile, a re-epithelization process in the intradermal was observed and this new epidermis generated hair follicles was detached.

In the developing embryo and hair follicle cycling, the formation of hair follicle is regulated by epithelial-morphogenesis interactions, and hair follicle regeneration relies on the signals derived from the dermis where contains the hair matrix and DP cells (Botchkarev and Kishimoto 2003). Although previous study suggested DP cells did not induce detectable hair follicles in the dermis, they could induce hair bud-like structures when implanted alone (Nilforoushzadeh et al. 2017). Those results applied that the presence of DP cells provided a device for a starting of hair follicle regeneration and additional DP cells implanted into dermis was not sufficient to induce hair generation (Leirós et al. 2014). Thus, the reconstruction of epithelial-morphogenesis interactions was very important for hair follicle forming. Compare with the data from the experiment which using the mixture of the epithelial cells and DP cells (Nilforoushzadeh et al. 2017), our data showed that injection of a mixture of ESCs and DP cells induce higher hair densities in nude mice. Those evidences show that the mixture of ESCs and DP cells are sufficient and efficient to induce hair generation, and ESCs are better than differentiated ones in the hair follicle regeneration (Dunnwald et al. 2001; Pellegrini et al. 1999).

In general, contracture and re-epithelialization result in wound closure and epithelization without epidermal appendage development leads to alopecia (Houschyar et al. 2015). Several studies suggested that progenitor epidermal cells are ideal seed cells in the generation of successful tissue engineered skin (Ito and Cotsarelis 2008). But Qi et al.’s experiments (2008) showed that a composite skin consisting of xenogeneic dermis, ESCs and DP cells promoted reconstruction of epidermis, unfortunately, without appendage development. Those evidences indicated that selected ideal stem cells sources did not ensure the persistence and function of the tissue engineered constructs. However, after grafting the skin constructs containing hair follicle stem cells and DP cells, hair bud-like structures in the remodeling dermis were observed in wound healing process (Leirós et al. 2014). Compare the data in detail with Qi et al.’s experiment (2008), maybe the different stem cells and their niches are responsible for the different outcome. Furthermore, Ito et al. (2007) found that the nascent follicles arise from epithelial cells in response to wound stimulus, and Jiang et al. (2010) observed that the activated epithelial cells by wounding could induce telogen to anagen transition. Based on this phenomenon, it is not surprised that injection of a mixture of ESCs or epithelial cells and DP cells into Nude Mice with full-thickness defect led to hair follicle regeneration (Wang et al. 2016). Because skin wound provides a critical influence of microenvironmental cues on cellular fate to regenerate hair follicle. Thus, the rearrangement of seed cells and their niches is essential and necessary for tissue-engineered construct success (Toyoshima et al. 2012).

In the current study, implantation of ESCs and DP cells complex regenerated a bioengineered skin, and those results strongly implied that fully functional skin construct could regenerate via rearrangement of seed stem cells and their niches. This view was partly supported by the process of androgenetic alopecia on the other side (Al-Refu 2012). Although the stem cells maintained in the bald scalp, epithelial stem cells under the influence of androgens, and the change of stem cells microenvironment, could not differentiate into progenitor cells. The number of bulb matrix cells diminished which was responsible for the diminutive hair follicle.

Although the epithelialization with epidermal appendage was observed in our experiment, we were not clear whether the remodeling dermis had the normal function. And further studies should be performed to assess the feature, and elucidate the mechanism by which stem cell niches will contribute to regeneration of skin.
